# A Sleepless Guardian

**Published:** 1875-12

**Authors:** 


### A SLEEPLESS GUARDIAN.

It is of a watchman whose sole duty is to give an alarm in case of fire, that we would speak. He takes no notice of riots or other disturbances. No noise moves him. Torch-light processions with bands of music may pass, but he never moves a muscle. His business is to watch for fire, and he sticks to it. When a fire comes, he speaks, and in no uncertain tones. The neighbors need no voice but his to convince them that there really is a small conflagration, which may become serious if it be not stopped.

We have a new neighbor in our Eighth street building, and we are a good deal interested in him. He is "The American Fire Detector Company." He makes and sells the wide-awake watchman just mentioned.

Mr. Charles D. Fredericks, the widely-known photographer, is the president of this corporation. He has contributed liberally of his capital for the development of this beautiful invention. The Fire Detector is a success, and is stationed as a permanent watchman in hundreds of edifices in this city.

The principle of its operation is simple, and as sure as simple. It is merely the natural expansion of a light strip of metal under the influence of heat. The tendency of a ribbon of metal, held at one end, and curved, is to straighten when exposed to heat. This is an unerring law of nature, and is never deviated from. This law is here taken advantage of by so adjusting the strip of metal that when the heat reaches a given height, a lever is released which connects with an alarm-bell, and creates a tremendous din. The instrument has a dial with figures denoting all probable degrees of temperature; and the indicator is placed at any degree which could be deemed to indicate danger. The instant the heat ascends to that height, the alarm sounds. It does not wait for the fire to attain headway and force, but summons help at the first approach of danger.

This little watchman has saved life and property already, as appears in evidence. Amelia Strauss asserts that this instrument in her house, No. 1027 Third Ave., unquestionably saved her life. Mr. T. H. Amidon, the well-known hatter of No. 250 Fifth Avenue certifies that the alarm given by the Detector on the occasion of an accidental fire in his engine-room aroused all the occupants of the house and thereby saved the premises. Had this vigilant guardian been on duty in the house of Mr. Jacob Stiner, the great tea merchant, at the time of the fire which destroyed his life, and that of his wife and daughter, that awful calamity would surely have been averted.

These Detectors are pretty little affairs, and look like a small clock. They are variously arranged and may be used as portable devices in the chief rooms having fires, or they may be distributed about as fixtures, all connected by concealed wires, with one enunciator in the hall. With such an arrangement no fire could possibly gain a dangerous headway without creating an alarm sufficient to startle all the inmates of the house, if not the near neighbors. Of course a fire discovered thus early could readily be quenched. Here is where the value of the Detector shows conspicuously, because it will usually announce a fire long before light or smoke enough is created to attract the attention of any human being.

We like this new neighbor of ours, and we want our friends to call and interview him. He is doing a noble work, and deserves success. He is the friend of the fire insurance people, because he saves them from heavy loss. He ought to be the friend of every man, for he is able to save life and property, by standing guard with sleepless vigilance, over the houses entrusted to his care. Let our friends who think they would like to station this watchman under their roof, write the company for descriptive pamphlets, or call and witness its operation for themselves. The cost of the Detector is moderate, and will fully pay for itself in the comfort and security gained.

---

An Analysis of one hundred and nineteen separate samples of ale and porter sold over the counter by publicans in various parts of London shows such a percentage of alcohol that it is obvious that a person who drinks two quarts of fourpenny ale or porter, consumes more alcohol than is contained in half a pint of brandy or whiskey. This will, no doubt astonish a good many people who are apt to think a couple of quarts of ale a day quite a moderate allowance, and when they find intoxication from beer among the lower classes so common, are apt to attribute it to some mysterious adulteration of beer and ale. The London *Sanitary Record*, however, says the main adulteration of ale and porter practised in London is the addition of sugar or treacle and water, and the lamentable frequency of intoxication is mainly due to excess of quantity rather than to defect in quality of beer.

